# Neurofeedback as neuroempowerment technique for affective regulation and interoceptive awareness in adolescence: preliminary considerations applied to a psychogenic pseudosyncope case

**DOI:** 10.3389/fresc.2023.1056972

**Published:** 2023-06-30

**Authors:** Michela Balconi, Laura Angioletti, Davide Crivelli

**Affiliations:** ^1^ International research center for Cognitive Applied Neuroscience (IrcCAN), Catholic University of the Sacred Heart, Milan, Italy; ^2^ Research Unit in Affective and Social Neuroscience, Department of Psychology, Catholic University of the Sacred Heart, Milan, Italy

**Keywords:** neurofeedback, biofeedback, affective regulation, adolescence, pseudosyncope, neurocognitive enhancement, neurorehabilitation

## Abstract

Revisions of classical models of acute stress response spectrum and defence cascade process might represent a valuable background for the interpretation of the link between affective reactions, traumatic experiences, and Psychogenic pseudosyncope (PPS) events in childhood and across the lifespan. Indeed, associations between subjective emotional life, early exposure to distressing and/or traumatic events, and PPS have fuelled a debate on potential causes of occurrence and recurrence of such a peculiar clinical manifestation. At the same time, such background suggests that empowering stress management and affective regulation skills could be the target for neurorehabilitation interventions aiming at reducing the severity of symptomatology and/or improving awareness and management of pseudosyncopal spells. Specifically, neuro/biofeedback-based empowerment of self-regulation skills, associated to an increased interoceptive increased awareness, could be a promising complement to classical psychological therapies. Starting from the presentation of a paediatric PPS clinical case, the present work discusses the relevance of assessing affective appraisal and autonomic reactivity in individuals suffering from PPS episodes and introduces a novel potential neuroempowerment protocol aimed at improving self-regulation and stress management skills in adolescence based on a combined neurofeedback and embodied-awareness intervention. By capitalizing available evidence of the effects of neuromodulation and embodied practices on self-awareness/regulation across the life-span, the proposed protocol is based on neurofeedback-supported affective management training, as well as both contemplative and informal awareness exercises devised to be appealing and challenging even for younger patients.

## 1. Introduction

Besides connoting adolescence as a critical developmental stage in the lifespan, the complex interaction between psychological evolution, main hormonal and physical changes, and both structural and functional neural development (1–3) also represents the unstable background in which many self-definition and self-determination processes delve their roots. During such period, identity, as well as self-awareness, metacognition, and executive control skills are challenged and, ultimately, mature (1, 2). While such a process holds the notable potential of flourishing and personal growth, its challenging and changeable nature also make the period of transition from childhood to adulthood peculiarly vulnerable. Adolescence is, indeed, associated with increased risk taking and dysfunctional behaviours (3, 4) and—it being the period when many psychopathological conditions set place and occurs (5, 6), or makes themselves evident—is deemed to be determinant for mental and physical wellbeing or distress later in life.

A primary and disruptive role in defining atypical vs. typical developmental trajectories is played by the exposure to distressing and/or traumatic events during such vulnerable period or during childhood, with subsequent exacerbation of the clinical sequelae of those negative events. Exposure to trauma in the early and mid steps of the lifespan can, indeed, have a pervasive impact on psychological, physical, and neural development, with a wide and robust evidence base highlighting its implication in far-ranging psychopathology, including depression, anxiety, suicide attempts, psychosis, substance use disorders, personality disorders, and self-regulatory disorders (7–9). Moreover, distressing/traumatic events have been associated to alterations in the development of brain structure and function, diminished cognitive functioning, dysfunctional control of behaviour, reduced ability to focus and sustain attention, impulsivity, and problems in interpersonal relationships and academic performance (10–14).

According to recent literature reviews, a further transdiagnostic features of atypical developmental trajectories might be the compound impairment of interoceptive sensitivity/awareness, dysregulation of physiological homeostasis and stress responses, and altered affective regulation (15–17). As an example, Zamariola and colleagues (18) have pointed out the strict relationship between interoceptive skills and affective regulation. According to the authors, people who have strong interoceptive abilities are better able to regulate their emotions. On the other hand, interoception influences emotion especially when physiological arousal is abnormal and differences between anticipated and experienced interoceptive signals may cause anxiety (19–21).

More specifically, the link between the ability to effectively process internal sensations and focus on internal states and proficiency in controlling behaviour and self-regulation has been stressed, with alterations in interoceptive awareness being associated with health conditions (22–24), depression (25), anxiety (19), eating disorders (26), and alexithymia (27). Also, it is here relevant to note that awareness for inner states and interoceptive signals was shown to play a relevant role in higher-order cognition, including learning and decision making (28), as well as processing, regulation, and memory of emotional stimuli (29, 30), ultimately influencing the development of relational and affective regulation ability (31). Affective regulation—understood as the ability to manage and modulate one's own emotional responses—represents itself a critical life skill, since it enables adaptation and flexibility of responses in emotionally-laden situations, and evolves across the lifespan (32). While in childhood, emotions are frequently expressed and managed with external support, during adolescence parental support is less valued and sought-for, yet adaptive internal emotion regulation is still limitedly efficient (33). Finally, in adulthood, internal regulatory strategies settle and become more and more efficient in management of affective experience (34).

## 2. Neurofeedback and neurocognitive enhancement of affective regulation

When facing affective regulation as a potential target for neurorehabilitation protocols, a first main challenge is posed by the complex interplay between conscious and unconscious processes that shapes the regulation of emotions and affects (35). The series of information-processing steps and the outcome of affective regulation are, indeed, built upon both subjective reflexive experience and conscious mechanisms—which modulate appraisal of the eliciting emotional stimulus and ongoing monitoring of subjective experience—and unaware automatic reactions and implicit information processing—which lead to basic approach-avoidance drives, physiological reactions (mainly interoceptive), and activation of automated response schemata to eliciting situation. Personal proficiency in orienting attention to such implicit processes and awareness of those automatic reactions and uncontrolled affective responses may foster the ability to de-automatize dysfunctional response patterns, and promote self-monitoring and regulation skills. Interventions aimed at empowering that type of awareness and modulation of own internal states and automatic reactions may then help to recover altered affective and self-regulation skills, as well as the ability to face negative or disruptive emotions in critical situations. Training those abilities might, in turn, prevent or limit the occurrence of unwanted uncontrolled pathological manifestations, such as disturbing thoughts and feelings, mental or physical distress bursts, externalizing or internalizing reaction symptoms, as well as functional neurologic symptom (e.g., psychogenic non-epileptic seizures, psychogenic pseudosyncope).

Entering the domain of neurocognitive enhancement (36), the therapeutic goal of reducing such pathological manifestations by improving latent affective regulation skills can be specifically pursued by combining traditional cognitive and behavioural techniques (e.g., visualization and embodied practices) with complementary psychophysiological intervention techniques (e.g., non-invasive brain stimulation or bio/neurofeedback (37, 38). Such combination is expected to make quicker and/or more effective the course of personalized rehabilitation, functional re-education or skills training.

Among psychophysiological techniques used to promote brain plasticity during neurocognitive enhancement and neurorehabilitation interventions, we propose that neurofeedback should be considered a quite promising one even when adolescents are the target of the intervention. Going down to specifics, neurofeedback is an applied psychophysiological technique which, by including the practitioner in a virtuous circle of implicit learning, primarily aims at:

(i) capturing and real-time monitoring the central physiological modifications (typically, electrophysiological activity) that accompany some mental states (such as distraction with respect to focused attention, or a state of excessive activation due to a dysfunctional management of the stress response);
(ii) strengthening the ability to become aware of such modifications;
(iii) and improving the ability to exercise intentional control over those mental states through the development of individual strategies and trough training of emotional self-regulation, attention regulation, and executive control skills.

Individual strategies are specifically developed to interrupt unwanted automatic reactions (such as a reaction of rejection and, then, black-out due to increased affective arousal in a specific situation) or lower their intensity or effect, and are created and strengthened during neurofeedback practice.

The neurofeedback technique pursues that threefold objective by, at first, accurately measuring one or more biosignals, understood as measurable physiological signals that mirror the fluctuation of the activity of a neural network or system. Such signals are, then, processed in real time via predetermined data processing pipelines and automated classifiers. Finally, the quantification of biosignal modulations is translated into easy-to-understand feedbacks (mostly visual and/or acoustic) that are presented to the practicers via dedicated software. Those feedbacks generally consist of sounds that change in intensity and/or tone, animated graphs that change according to physiological activations, moving animations or videogames that adapts and responds to changes in bodily activity. Going down to specifics, the feedbacks provided by the software managing the neurofeedback system (them being simple graphs, tones or other acoustic stimuli, or even complex animations/games) are typically devised to linearly mirror the modulation of a targeted biosignal (e.g., the power of alpha oscillations, the a relative power of theta oscillations with respect to alpha, the coherence of alpha activity over lateralized prefrontal regions), so that any electrophysiological is recorded and used to modulate rewards and punishments that foster implicit learning. In addition, it is worth noting that thresholds for proving rewarding and/or punishing feedbacks can be both set manually and actively controlled by the trainer, or set to be automatically managed by the software, which, in such cases, would autonomously adapt them to keep a constant reward rate depending on the actual real-time practicer's performance.

The intrinsic advantage of this technique consists in the degree of active involvement and agentive commitment required to the trainee in order for the technique to fully express its potential. The enhancement of cognitive-affective abilities and related neuroplastic changes that are pursued by practice are, indeed, reached by training the sensitivity of monitoring and active control of the neurofunctional substrate of those trained abilities. To reach such goal, neurofeedback requires the intentional commitment and active involvement of the practitioner, as well as a minimal degree of metacognitive and self-reflection skills. Those operational aspects of the neurofeedback technique become particularly valuable when focusing on adolescents as targets of the intervention. They might, indeed, foster self-efficacy while taking advantage of the intrinsic drive towards self-determination and expression of an actual agentive role that connote such period of life.

Neurofeedback systems thus provide practicers with real-time information on the modulation of their physiological activation states or their neurofunctional activity at rest or during the execution of a task. Audio-visual feedbacks act as the fuel for implicit learning processes and for consolidation of individual strategies developed to try and control those states and those physiological correlates of self-regulation processes. By exploiting the principles of operant conditioning, in fact, the neurofeedback systems return positive (i.e., reinforcements) and negative (i.e., punishments) feedback on the progress of mental training to the practicer, thus giving him/her the opportunity to directly evaluate the effect of their own efforts and gain awareness even on that self-enhancement process. The virtuous cycle created by neurofeedback systems is thus made up of four main interdependent elements, namely

(i) the central physiological modifications associated with a task (e.g. progressive modulation of alpha power during an interoceptive attentiveness or relaxation task),
(ii) the presence of an external feedback provided by the system (e.g. the modulation of an acoustic feedback mirroring those central physiological modifications),
(iii) an implicit associative learning process (i.e. the pre-reflexive process of associating external feedbacks to physiological modulations, and then implicitly using such knowledge to try and regulate physiological activity using the external feedback as a cue),
(iv) and the consequent consolidation of trained cognitive or affective regulation functions (i.e. the transfer of such implicitly learned skills to aware control, so to define and strengthen individual strategies to actively monitor and regulate cognitive-affective processes),

which animate and consolidate the empowerment process towards greater self-awareness and greater self-regulation skills.

Relevant to the application of the neurofeedback technique for enhancement and rehabilitation of affective regulation, it has been suggested that the specific potential of such a technique to make even minimal modifications of the neurofunctional correlates of trained mental processes easily perceptible may contribute to the learning process and maintenance of training effects (35, 39). According to models of implicit learning (35, 40), gradual and self-paced unveiling of pieces of information that would otherwise remain inaccessible to the conscience can, indeed, promote greater awareness and a greater sense of agency in learning, growth, and empowerment paths, thus fostering the efficacy of such processes of personal change. That because practitioners are directly involved, with the support of the trainer, in the metacognitive process of finding and consolidating personal strategies to consciously note and intentionally modify their levels of neurophysiological activation, e.g., using specific visualizations, self-talk, or relaxation/focusing exercises to down-regulate maladaptive stress responses, up-regulate executive control activity, or disengage from dysfunctionally salient stimuli.

Technically speaking, in neurofeedback protocols, one or two sensors are typically used to collect information on the electrophysiological correlates of cognitive activity and return this information to the practitioner. The recording system amplifies and transfers the biosignals detected by the sensors to a software, which finally converts collected pieces of information into simple positive or negative feedback or, as is the case in the most recent devices, uses them to control videogames designed to train specific functions—such as focused or sustained attention skills, adaptive relaxation, or up/downregulation of emotion processing systems (for further technical and methodological information, see (39, 41, 42). The duration of the training varies according to its goals (e.g., enhancing control over emotional reactions or increasing the relaxation and stress management skills) and according to the resources of the practitioner, but the typical session lasts about 45–60 min including 20-to-40 min of actual practice with the device. In addition, the importance of customizing activities and calibrating the systems for detecting and providing personalized feedbacks to the individual practitioner is now widely recognized. Neurofeedback protocols must, therefore, always be preceded by psychological and psychophysiological assessment sessions aimed at defining a complete and reliable picture of the physiological characteristics—as well as of the strengths and weaknesses of the psychological and cognitive-affective profile—of the person who will be involved in the training, sketching his/her subjective starting point. Such peculiar attention towards assessment and personalization of interventions becomes particularly important when planning to use the technique to enhance affective regulation skill and when working with still developing users. Indeed, in those cases there is often no unique organic or functional deficit (e.g., a cognitive deficit due to lesion of the anterior regions of the cerebral cortex) that can clearly guide the design of an intervention protocol. And again, in such cases the aims of the intervention protocol must be contextualized with respect to the profile of individual skills and weaknesses, keeping in mind the variability and potential evolution of personal skills within and between persons that are going through the changing valley of adolescence and whose developmental trajectories may have been marked by early distressing or traumatic events.

## 3. Psychogenic pseudosyncope as an exemplifying case

Trying to cross the bridge between methodological discussion and actual practice, we will now briefly present a case of suspect functional neurological symptom disorder—namely, psychogenic pseudosyncope—in early adolescence, as the starting point to define an exemplifying neurofeedback-based intervention protocol.

A, a 13-year-old female, was reported to the cardiology unit of her local hospital after having presented a remarkable increase of pre-syncopal and syncopal events over a short period. Anamnestic data collection highlighted that the primary triggers of A's syncopal events were light head concussions, pain, heat, and affective experiences (social stress and strong emotion reactions). All episodes occurred in the presence of witnesses, in social contexts and mostly at school. In none of them, the suspected syncopal event led to contusion or physical trauma from the fall. No evidence for actual loss of movement control or consciousness, nor for post-ictal confusion or drowsiness, was provided. ECG monitoring during the events highlighted normal cardiac responses and mild tachycardia. Following specialist clinical and instrumental examinations, A's clinical picture has been attributed to a form of suspected psychogenic pseudosyncope (PPS).

Both clinical observations and cohort studies highlighted that PPS and medically unexplained syncopal events are often associated to distressing situations and emotional triggers (43–46). Consistently, it was reported that individuals suffering from PPS episodes typically present dysfunctional affective regulation skills (especially when facing negatively-valenced events) and autonomic dysregulation in the sympathetic component (43, 47, 48). Evidence from psychiatry suggests that most cases of PPS represent a conversion disorder (49) and it has been suggested that exposure to traumatic events, overwhelming distress, and adverse early life experiences might act—just as they do in case of other psychogenic disorders—as predisposing factors for the development of syncopal and pseudosyncopal events during childhood or later in life (43, 50, 51).

An alteration of psychophysiological responsiveness to emotional stimuli was observed even in A during standardized psychophysiological assessment (52). Namely, A showed heightened autonomic reactivity when presented with emotion-laden visual stimuli (especially negative-valenced ones), paired with heightened levels of anxiety and depressive symptomatology, heightened sensitivity to positive rewards, and restrained self-perception of arousal when presented with positive high-arousal images.

Based on that emerging psychophysiological profile, empowering aware affective regulation skills and enhancing neurofunctional structures supporting regulation of emotion and psychophysiological reactions could be deemed a primary intervention target. Available literature on neurofeedback interventions on affective regulation mainly grounds on the dual prefrontal system model of emotion processing (53–56), which posits the existence of separate systems involving left and right prefrontal structures, respectively supporting positive affects or approach drives and negative affects or avoidance drives. Specifically, the profile of anxiety/depression, enhanced sensitivity to rewards, and altered affective experiences is consistent with a potential imbalance towards left vs. right prefrontal system activity (57). The above-mentioned enhancement targets could be pursued by implementing a gamified dual sensor neurofeedback protocol with bilateral frontal montage in F3 and F4 electrode positions (39, 42), rewarding a balanced coherent EEG activity between left/right areas (electrophysiological measure for feedback management: alpha band coherence over F3/4 electrode sites). Reward thresholds should be calibrated on the practicer's electrophysiological response profile in the alpha EEG band after a preliminary psychophysiological assessment including, as example of metrics, log-transformed alpha asymmetry at rest, individual alpha peak frequency, and bilateral prefrontal alpha coherence at rest. Then, the training schedule could be devised so to include a first part aimed at improving self-monitoring and modulation of asymmetrical prefrontal activations at rest (intensive training: three sessions a week) and a second part where the practicer is asked to exercise while being presented with emotional stimuli (paying, in this case, peculiar attention to negative ones), gradually reducing the frequency of weekly sessions from two to one but implementing the practice with metacognitive and self-awareness exercises (e.g., journaling, self-observation) to be performed autonomously and discussed in at the weekly appointments. Such kind of intervention protocols aims at training the practicer ability to recognize the mindset associated to altered affective reactivity and then activate conscious appraisal processes and self-regulation mechanisms to modulate dysfunctionally enhanced activity of the left prefrontal system over the right one. In order to consolidate learning outcomes and foster self-regulation/efficacy skills, combining the neurofeedback protocol with simple parallel self/bodily-awareness and metacognitive exercises is advisable.

## 4. Conclusions

Since initial applications of the neurofeedback technique in experimental and clinical context (58–60), its application have extended, among other conditions, to anxiety disorders, autism spectrum disorders, addiction, re-education following brain injury, and impairment of affective regulation. The latest extensive critical reviews of the empirical evidence on the efficacy of neurofeedback interventions have highlighted how the technique might be effective in increasing emotion regulation even in childhood/adolescence, although further systematic research is needed to properly assess the extent and limits of the potential of this intervention technique with paediatric users (see 61, 62).

More recently, however, research has begun to show interesting results with respect to the enhancement of stress management and affective regulation when using an integrated approach that combines neurofeedback technique with mental training based on mindfulness approach and body awareness (37, 38, 63–67). In fact, a growing literature has reported how supporting mindfulness-based training with neurofeedback systems—able to foster awareness of physiological changes that accompany the practice—allows improving attention and emotional regulation skills, as mirrored by decreased levels of anxiety and perceived stress, increased mental energy, and optimization of the use of cognitive resources, via intensive protocols in young adults.

Therefore, it is necessary to consider the role that the neurofeedback technique assumes in the patient's ability to develop a greater awareness of his own affective and interoceptive correlates, as a discriminating element for the ability to understand his/her own emotional states and modulate them, especially in conditions of emotional dysregulation. Building on available evidence, we suggest that the efficacy of combined mindfulness-neurofeedback protocols in atypically developing children and adolescents presenting affective regulation impairments (including, but not limited to, functional neurological symptom disorders such as psychogenic pseudosyncope) could be worth of systematic empirical testing, so to properly assess their potential with such populations and for such therapeutic challenges over *a priori* expectations. Such neurocognitive enhancement protocols, given their sustainable nature and due to their easy integration with other complementing neurorehabilitation interventions, might be a promising supportive therapy.

To sum up, yet immature affective regulation strategies in adolescence, together with still evolving higher-order cognitive, self-awareness, and emotion appraisal skills, makes the impact of distressing and/or traumatic events in this period a substantial risk factor for psychopathology and atypical development, with both neurocognitive and psychiatric sequelae that should become targets for personalized rehabilitation interventions.

## Data Availability

The original contributions presented in the study are included in the article/Supplementary Material, further inquiries can be directed to the corresponding author.

## References

[1] Blakemore S-J Choudhury S. Development of the adolescent brain: implications for executive function and social cognition. J Child Psychol Psychiatry. (2006) 47:296–312. 10.1111/j.1469-7610.2006.01611.x 16492261

[2] Coleman JC. The nature of adolescence. London: Routledge (2010). 10.4324/9780203805633

[3] Crone EA van Duijvenvoorde ACK Peper JS. Annual research review: neural contributions to risk-taking in adolescence–developmental changes and individual differences. J Child Psychol Psychiatry. (2016) 57:353–68. 10.1111/jcpp.12502 26889896

[4] Steinberg L. Risk taking in adolescence: new perspectives from brain and behavioral science. Curr Dir Psychol Sci. (2007) 16:55–9. 10.1111/j.1467-8721.2007.00475.x

[5] Kessler RC Berglund P Demler O Jin R Merikangas KR Walters EE. Lifetime prevalence and age-of-onset distributions of DSM-IV disorders in the national comorbidity survey replication. Arch Gen Psychiatry. (2005) 62:593–602. 10.1001/archpsyc.62.6.593 15939837

[6] Paus T Keshavan M Giedd JN. Why do many psychiatric disorders emerge during adolescence? Nat Rev Neurosci. (2008) 9:947–57. 10.1038/nrn2513 19002191 PMC2762785

[7] Green JG McLaughlin KA Berglund PA Gruber MJ Sampson NA Zaslavsky AM Childhood adversities and adult psychiatric disorders in the national comorbidity survey replication I. Arch Gen Psychiatry. (2010) 67:113. 10.1001/archgenpsychiatry.2009.186 20124111 PMC2822662

[8] D’Andrea W Ford J Stolbach B Spinazzola J van der Kolk BA. Understanding interpersonal trauma in children: why we need a developmentally appropriate trauma diagnosis. Am J Orthopsychiatry. (2012) 82:187–200. 10.1111/j.1939-0025.2012.01154.x 22506521

[9] Lippard ETC Nemeroff CB. The devastating clinical consequences of child abuse and neglect: increased disease vulnerability and poor treatment response in mood disorders. Am J Psychiatry. (2020) 177:20–36. 10.1176/appi.ajp.2019.19010020 31537091 PMC6939135

[10] Holtmann M Buchmann AF Esser G Schmidt MH Banaschewski T Laucht M. The child behavior checklist-dysregulation profile predicts substance use, suicidality, and functional impairment: a longitudinal analysis. J Child Psychol Psychiatry. (2011) 52:139–47. 10.1111/j.1469-7610.2010.02309.x 20854363

[11] Teicher MH Samson JA. Annual research review: enduring neurobiological effects of childhood abuse and neglect. J Child Psychol Psychiatry. (2016) 57:241–66. 10.1111/jcpp.12507 26831814 PMC4760853

[12] Zeanah CH Humphreys KL. Child abuse and neglect. J Am Acad Child Adolesc Psychiatry. (2018) 57:637–44. 10.1016/j.jaac.2018.06.007 30196867 PMC6615750

[13] Heim CM Entringer S Buss C. Translating basic research knowledge on the biological embedding of early-life stress into novel approaches for the developmental programming of lifelong health. Psychoneuroendocrinology. (2019) 105:123–37. 10.1016/j.psyneuen.2018.12.011 30578047 PMC6561839

[14] van Der Kolk B Ford JD Spinazzola J. Comorbidity of developmental trauma disorder (DTD) and post-traumatic stress disorder: findings from the DTD field trial. Eur J Psychotraumatol. (2019) 10:1562841. 10.1080/20008198.2018.1562841 30728917 PMC6352932

[15] Heleniak C Jenness JL Vander Stoep A McCauley E McLaughlin KA. Childhood maltreatment exposure and disruptions in emotion regulation: a transdiagnostic pathway to adolescent internalizing and externalizing psychopathology. Cognit Ther Res. (2016) 40:394–415. 10.1007/s10608-015-9735-z 27695145 PMC5042349

[16] Murphy J Brewer R Catmur C Bird G. Interoception and psychopathology: a developmental neuroscience perspective. Dev Cogn Neurosci. (2017) 23:45–56. 10.1016/j.dcn.2016.12.006 28081519 PMC6987654

[17] Erwin MC Mitchell MA Contractor AA Dranger P Charak R Elhai JD. The relationship between distress tolerance regulation, counterfactual rumination, and PTSD symptom clusters. Compr Psychiatry. (2018) 82:133–40. 10.1016/j.comppsych.2018.01.012 29477706

[18] Zamariola G Frost N Van Oost A Corneille O Luminet O. Relationship between interoception and emotion regulation: new evidence from mixed methods. J Affect Disord. (2019) 246:480–5. 10.1016/j.jad.2018.12.101 30599372

[19] Paulus MP Stein MB. An insular view of anxiety. Biol Psychiatry. (2006) 60:383–7. 10.1016/j.biopsych.2006.03.042 16780813

[20] Garfinkel SN Tiley C O’Keeffe S Harrison NA Seth AK Critchley HD. Discrepancies between dimensions of interoception in autism: implications for emotion and anxiety. Biol Psychol. (2016) 114:117–26. 10.1016/j.biopsycho.2015.12.003 26724504

[21] Owens AP Allen M Ondobaka S Friston KJ. Interoceptive inference: from computational neuroscience to clinic. Neurosci Biobehav Rev. (2018) 90:174–83. 10.1016/j.neubiorev.2018.04.017 29694845

[22] Khalsa SS Adolphs R Cameron OG Critchley HD Davenport PW Feinstein JS Interoception and mental health: a roadmap. Biol Psychiatry Cogn Neurosci Neuroimaging. (2018) 3:501–13. 10.1016/j.bpsc.2017.12.004 29884281 PMC6054486

[23] Quadt L Critchley HD Garfinkel SN. The neurobiology of interoception in health and disease. Ann N Y Acad Sci. (2018) 1428:112–28. 10.1111/nyas.13915 29974959

[24] Angioletti L Balconi M. Interoceptive attentiveness and autonomic reactivity in pain observation. Somatosens Mot Res. (2022) 39:81–9. 10.1080/08990220.2021.2005016 34847833

[25] Harshaw C. Interoceptive dysfunction: toward an integrated framework for understanding somatic and affective disturbance in depression. Psychol Bull. (2015) 141:311–63. 10.1037/a0038101 25365763 PMC4346391

[26] Pollatos O Kurz A-L Albrecht J Schreder T Kleemann AM Schöpf V Reduced perception of bodily signals in anorexia nervosa. Eat Behav. (2008) 9:381–8. 10.1016/j.eatbeh.2008.02.001 18928900

[27] Herbert BM Pollatos O. The body in the mind: on the relationship between interoception and embodiment. Top Cogn Sci. (2012) 4:692–704. 10.1111/j.1756-8765.2012.01189.x 22389201

[28] Werner NS Jung K Duschek S Schandry R. Enhanced cardiac perception is associated with benefits in decision-making. Psychophysiology. (2009) 46:1123–9. 10.1111/j.1469-8986.2009.00855.x 19558399

[29] Pollatos O Schandry R. Emotional processing and emotional memory are modulated by interoceptive awareness. Cogn Emot. (2008) 22:272–87. 10.1080/02699930701357535

[30] Füstös J Gramann K Herbert BM Pollatos O. On the embodiment of emotion regulation: interoceptive awareness facilitates reappraisal. Soc Cogn Affect Neurosci. (2013) 8:911–7. 10.1093/scan/nss089 22933520 PMC3831556

[31] Quattrocki E Friston K. Autism, oxytocin and interoception. Neurosci Biobehav Rev. (2014) 47:410–30. 10.1016/j.neubiorev.2014.09.012 25277283 PMC4726659

[32] Young KS Sandman CF Craske MG. Positive and negative emotion regulation in adolescence: links to anxiety and depression. Brain Sci. (2019) 9:76. 10.3390/brainsci9040076 30934877 PMC6523365

[33] Zimmermann P Iwanski A. Emotion regulation from early adolescence to emerging adulthood and middle adulthood: age differences, gender differences, and emotion-specific developmental variations. Int J Behav Dev. (2014) 38:182–94. 10.1177/0165025413515405

[34] Gross JJ. Emotion regulation in adulthood: timing is everything. Curr Dir Psychol Sci. (2001) 10:214–9. 10.1111/1467-8721.00152

[35] Balconi M Fronda G Venturella I Crivelli D. Conscious, pre-conscious and unconscious mechanisms in emotional behaviour. Some applications to the mindfulness approach with wearable devices. Appl Sci. (2017) 7:1280. 10.3390/app7121280

[36] Balconi M Crivelli D. Wearable devices for self-enhancement and improvement of plasticity: Effects on neurocognitive efficiency. In: Esposito A Faundez-Zanuy M Morabito FC Pasero E, editors. Quantifying and processing biomedical and behavioral signals smart innovation, systems and technologies. Cham: Springer International Publishing (2019). p. 11–22. 10.1007/978-3-319-95095-2_2

[37] Balconi M Fronda G Crivelli D. Effects of technology-mediated mindfulness practice on stress: psychophysiological and self-report measures. Stress. (2019) 22:200–9. 10.1080/10253890.2018.1531845 30472901

[38] Crivelli D Fronda G Venturella I Balconi M. Supporting mindfulness practices with brain-sensing devices. Cognitive and electrophysiological evidences. Mindfulness (N Y). (2019c) 10:301–11. 10.1007/s12671-018-0975-3

[39] Gruzelier JH. EEG-neurofeedback for optimising performance. III: a review of methodological and theoretical considerations. Neurosci Biobehav Rev. (2014b) 44:159–82. 10.1016/j.neubiorev.2014.03.015 24690579

[40] Cleeremans A Jiménez L. Implicit learning and consciousness: A graded, dynamic perspective. In: French RM Cleeremans A, editors. Implicit learning and consciousness: An empirical, philosophical and computational consensus in the making. Hove: Psychology Press (2002). p. 1–40.

[41] Budzynski T. H. ed. Introduction to quantitative EEG and neurofeedback. Advanced theory and applications. Burlington, MA: Elsevier Academic Press (2009). p. 1–549.

[42] Hammond DC Bodenhamer-Davis G Gluck G Stokes D Harper SH Trudeau D Standards of practice for neurofeedback and neurotherapy: a position paper of the international society for neurofeedback & research. J Neurother. (2011) 15:54–64. 10.1080/10874208.2010.545760

[43] Tannemaat MR van Niekerk J Reijntjes RH Thijs RD Sutton R van Dijk JG. The semiology of tilt-induced psychogenic pseudosyncope. Neurology. (2013) 81:752–8. 10.1212/WNL.0b013e3182a1aa88 23873974 PMC3776458

[44] van Dijk JG Wieling W. Pathophysiological basis of syncope and neurological conditions that mimic syncope. Prog Cardiovasc Dis. (2013) 55:345–56. 10.1016/j.pcad.2012.10.016 23472770

[45] Brignole M Moya A de Lange FJ Deharo J-C Elliott PM Fanciulli A 2018 ESC guidelines for the diagnosis and management of syncope. Eur Heart J. (2018) 39:1883–948. 10.1093/eurheartj/ehy037 29562304

[46] Crivelli D Balconi M. Trauma and syncope: which relationship do they share? Maltrattamento e Abus All’Infanzia. (2020b) 22(1):35–54. 10.3280/MAL2020-001004

[47] Donadio V Liguori R Elam M Karlsson T Montagna P Cortelli P Arousal elicits exaggerated inhibition of sympathetic nerve activity in phobic syncope patients. Brain. (2007) 130:1653–62. 10.1093/brain/awm037 17395613

[48] Buodo G Sarlo M Poli S Giada F Madalosso M Rossi C Emotional anticipation rather than processing is altered in patients with vasovagal syncope. Clin Neurophysiol. (2012) 123:1319–27. 10.1016/j.clinph.2011.12.003 22217960

[49] Raj V Rowe AA Fleisch SB Paranjape SY Arain AM Nicolson SE. Psychogenic pseudosyncope: diagnosis and management. Auton Neurosci. (2014) 184:66–72. 10.1016/j.autneu.2014.05.003 24882462

[50] Emiroglu FNI Kurul S Akay A Miral S Dirik E. Assessment of child neurology outpatients with headache, dizziness, and fainting. J Child Neurol. (2004) 19:332–6. 10.1177/088307380401900505 15224706

[51] O’Hare C McCrory C O’Leary N O’Brien H Kenny RA. Childhood trauma and lifetime syncope burden among older adults. J Psychosom Res. (2017) 97:63–9. 10.1016/j.jpsychores.2017.03.019 28606501

[52] Crivelli D Balconi M. Assessing physiological autonomic reactivity and appraisal in psychogenic pseudosyncope: preliminary evidence from a pilot study with two single-cases. Maltrattamento e Abus All’Infanzia. (2020a) 22(1):55–65. 10.3280/MAL2020-001005

[53] Gray J. A. (1990). Brain systems that mediate both emotion and cognition. Cogn Emot. 4, 269–88. Available at: http://www.informaworld.com/10.1080/02699939008410799 10.1080/02699939008410799

[54] Davidson RJ. Anterior cerebral asymmetry and the nature of emotion. Brain Cogn. (1992) 20:125–51. 10.1016/0278-2626(92)90065-T 1389117

[55] Balconi M Mazza G. Lateralisation effect in comprehension of emotional facial expression: a comparison between EEG alpha band power and behavioural inhibition (BIS) and activation (BAS) systems. Laterality. (2009) 15:361–84. 10.1080/13576500902886056 19536685

[56] Harmon-Jones E Gable Pa Peterson CK. The role of asymmetric frontal cortical activity in emotion-related phenomena: a review and update. Biol Psychol. (2010) 84:451–62. 10.1016/j.biopsycho.2009.08.010 19733618

[57] Gray JA. The neuropsychology of anxiety: An enquiry into the functions of the septo-hippocampal system. 2nd ed. New York: Oxford University Press (2003).

[58] Kamiya J. Conscious control of brain waves. Psychol Today. (1968) 1:56–60.

[59] Sterman MB Macdonald LR Stone RK. Biofeedback training of the sensorimotor electroencephalogram rhythm in man: effects on epilepsy. Epilepsia. (1974) 15:395–416. 10.1111/j.1528-1157.1974.tb04016.x 4527675

[60] Lubar JF Shouse MN. EEG And behavioral changes in a hyperkinetic child concurrent with training of the sensorimotor rhythm (SMR)—a preliminary report. Biofeedback Self Regul. (1976) 1:293–306. 10.1007/BF01001170 990355

[61] Yucha C Montgomery D. Evidence-based practice in biofeedback and neurofeedback.. Wheat Ridge, CO: Association for Applied Psychophysiology and Biofeedback (2008).

[62] Gruzelier JH. EEG-neurofeedback for optimising performance. I: a review of cognitive and affective outcome in healthy participants. Neurosci Biobehav Rev. (2014a) 44:124–41. 10.1016/j.neubiorev.2013.09.015 24125857

[63] Bhayee S Tomaszewski P Lee DH Moffat G Pino L Moreno S Attentional and affective consequences of technology supported mindfulness training: a randomised, active control, efficacy trial. BMC Psychol. (2016) 4:60. 10.1186/s40359-016-0168-6 27894358 PMC5127005

[64] Crivelli D Fronda G Balconi M. Neurocognitive enhancement effects of combined mindfulness–neurofeedback training in sport. Neuroscience. (2019a) 412:83–93. 10.1016/j.neuroscience.2019.05.066 31195055

[65] Crivelli D Fronda G Venturella I Balconi M. Stress and neurocognitive efficiency in managerial contexts: a study on technology-mediatedmindfulness practice. Int J Work Heal Manag. (2019b) 12:42–56. 10.1108/IJWHM-07-2018-0095

[66] Dunham CM Burger AL Hileman BM Chance EA Hutchinson AE Kohli CM Brainwave self-regulation during bispectral IndexTM neurofeedback in trauma center nurses and physicians after receiving mindfulness instructions. Front Psychol. (2019) 10:2153. 10.3389/fpsyg.2019.02153 31616348 PMC6775210

[67] Hunkin H King DL Zajac IT. EEG Neurofeedback during focused attention meditation: effects on state mindfulness and meditation experiences. Mindfulness (N Y). (2021) 12:841–51. 10.1007/s12671-020-01541-0

